# No difference in genotype frequencies of polymorphisms of the nitric oxide pathway between Caucasian normal and high tension glaucoma patients

**Published:** 2012-08-07

**Authors:** Johanna Weiss, Stephan A. Fränkl, Josef Flammer, Matthias C. Grieshaber, Gabor Hollo, Barbara Teuchner, Walter Emil Haefeli

**Affiliations:** 1 Department of Clinical Pharmacology and Pharmacoepidemiology University of Heidelberg, Heidelberg, Germany; 2 Eye Clinic of the University Hospital Basel, Basel, Switzerland; 3 Department of Ophthalmology, Semmelweis University, Budapest, Hungary; 4 Department of Ophthalmology, Medical University Innsbruck, Innsbruck, Austria

## Abstract

**Purpose:**

Substantial evidence suggests that ocular perfusion is regulated by nitric oxide (NO), and polymorphisms in genes encoding for enzymes involved in NO formation and degradation (endothelial nitric oxide synthase [*NOS3*] and cytochrome b-235 alpha polypeptide gene [*CYBA*]) might contribute to vascular dysregulation observed in glaucoma. We therefore assessed the association of glaucoma with polymorphisms of *NOS3* and *CYBA* previously associated with cardiovascular disease. We also compared the distribution of these polymorphisms in patients with high tension glaucoma (HTG) and normal tension glaucoma (NTG) and evaluated its association with vascular dysregulation in a subset of glaucoma patients.

**Methods:**

Three hundred Caucasian patients with HTG and 127 with NTG were enrolled in the study and genotyped for G894T (rs1799983) and T-786C (rs2070744) in *NOS3* and C242T (rs4673) in *CYBA*.

**Results:**

None of these polymorphisms had a different allele or genotype distribution between HTG and NTG patients nor had the presence of vasospasms any impact.

**Conclusions:**

We studied the frequencies of a set of relevant polymorphisms of the NO system in a large cohort of glaucoma patients and found no association. These results therefore suggest the absence of a relevant relationship with different glaucoma forms in Caucasians.

## Introduction

Open-angle glaucoma (OAG) is a group of neurodegenerative diseases resulting in loss of retinal ganglion cells and tissue remodeling of the optic nerve head. Clinically, the diagnosis of open-angle glaucoma is based on characteristic glaucomatous neuroretinal rim loss and corresponding visual field deterioration in the presence of an open chamber angle [1]. OAG has clinically been divided into two major primary forms: high tension glaucoma (HTG) in which intraocular pressure (IOP) is clinically significantly elevated and represents a strong risk factor for development and progression of glaucoma, and normal tension glaucoma (NTG), in which IOP is consistently within the statistically normal range [1,2]. In HTG, which is the most common form of open-angle glaucoma [3,4] dysfunction of the trabecular meshwork cells reduces trabeculocanalicular outflow of aqueous humor, and leads to significant elevation of IOP [5]. In contrast, IOP elevation is by definition not present in NTG. The pathophysiology of NTG regarding the development and progression of neuroretinal rim loss is presumed to be partially different [1,2]. Altered vascular regulation is frequently present both in HTG and NTG [2,6]. The dysfunction of the ciliary artery vascular endothelium and the decrease of ocular perfusion pressure lead to increased rate of retinal ganglion cell apoptosis in both glaucoma types [2,6].Vascular dysregulation in open-angle glaucoma, however, is not an isolated ocular alteration but a systemic feature which can be investigated also with non-ocular clinical methods [2,7,8]. Vascular dysregulation may precede the development of glaucomatous structural and functional damage, which suggests its pathophysiological relevance in the development of glaucoma [2]. Endothelin-1 (ET-1) is potent vasoconstrictor, which may lead to vasospasm even in healthy people [9]. In the eye, ET-1, and other circulating hormones have a major impact on the circulation as there is no strong blood-vessel barrier [10]. In case of an increased level of ET-1, the blood flow is reduced, which has been demonstrated in patients with OAG [11].

Nitric oxide (NO) is an active biologic agent involved in diverse physiologic processes. NO is synthesized from the amino acid L-arginine by NO synthase (NOS). While several NOS isoforms have been identified, circulating NO in the retina has been demonstrated to be synthesized solely in the vascular endothelium by the action of endothelial nitric oxide synthase (eNOS, NOS3) [12]. Several polymorphisms of *NOS3* are associated with reduced vascular NO levels and cardiovascular morbidity [13]. Among them are the G894T (Glu298Asp, rs1799983) and the T-786C (rs2070744) polymorphisms.

NO is degraded by several reactive oxidative species (e.g., superoxide anion) limiting its duration of action [14]. In the vascular wall superoxide anion is formed by the nicotinamide adenine dinucleotide phosphate (NAD(P)H) oxidase system [15,16] of which p22phox is an important subunit [17]. The C242T (Tyr72His, rs4673) polymorphism in the cytochrome b-235 alpha polypeptide gene (*CYBA*) encoding for p22phox reduces the vascular NAD(P)H oxidase activity thus diminishing local superoxide anion formation and increasing NO availability [17]. Studies addressing the association of the C242T polymorphism with vascular disease have reported conflicting results [18-25] suggesting that the impact of *CYBA* polymorphisms might be modulated by other factors one of which could be the generation of NO.

The vasodilator properties of NO may also play a major role in the regulation of ocular perfusion and polymorphisms in the *NOS3* and *CYBA* might contribute to vascular dysregulation observed in glaucoma. There is evidence that constant formation of nitric oxide (NO) by the endothelial and neuronal isoforms of the enzyme NO synthase (NOS) provides the maintenance of a basal vasodilator tone in the optic nerve head [26,27], which is a precondition of sufficient blood supply in this tissue. Further, systemic infusion of NO inhibitors reduces choroidal blood flow in animals and humans [27-30]. A clinical study investigated previously the ocular blood flow response to systemic nitric oxide synthase inhibition and found that an abnormal NO system in the optic nerve head and in the choroid in patients with glaucoma as compared with healthy controls based on a significantly less pronounced decrease of optic nerve head blood flow and fundus pulsation amplitude during nitric oxide synthase inhibition compared to healthy control subjects [31].

However, in Caucasians there are only three studies on the impact of *NOS3* polymorphism in primary open-angle glaucoma (POAG) and their results are conflicting [32-34]. Moreover, so far there are no data available on the potential role of the C242T polymorphism for the development of glaucoma.

We therefore aimed at assessing the relationship between glaucoma and the G894T and T-786C polymorphisms of *NOS3* and the C242T *CYBA* polymorphisms in a Caucasian cohort. Also, we compared the distribution of these polymorphisms in patients with HTG and NTG and evaluated its association with vascular dysregulation in a subset of glaucoma patients.

## Methods

### Patients and design

Patients were recruited in three different European cities at large tertiary care centers. All patients were Caucasians, consecutively enrolled when they came for a regular examination. All patients had advanced disease assessed morphologically by the C/D ratio (=0.7). There was no difference regarding the C/D ratio between patients with normal and high tension glaucoma (p>0.2, two-tailed *t*-test). The study protocol was approved by the responsible Ethics Committees of the participating institutions (Medical Faculty of the University of Basel, the University of Innsbruck, and the University of Budapest), and all participants provided informed consent for DNA analysis.

### Diagnosis of normal and high tension glaucoma

Patients with glaucoma met the following inclusion criteria: reproducible glaucomatous visual field damage in either eye in at least three successive perimetric tests, glaucomatous neuroretinal rim loss, and the absence of other optic neuropathies. Patients with IOP consistently >21 mmHg were diagnosed with HTG. In NTG patients IOP was consistently <21 mmHg, and the highest ever measured IOP was ≤21 mmHg.

Patients with any of the following criteria were excluded from in the study: history of other ocular or systemic disease (e.g., diabetes mellitus), current smoking, drug or alcohol abuse, ocular trauma, ocular infection, or ocular inflammation.

Assessment of vascular dysregulation included examination of extremities, color duplex sonography, laser Doppler flowmetry, and capillary microscopy. At least two positive test results, or the presence of cold extremities and one positive test result were required for a diagnosis of vascular dysregulation [8]. This was done only in a subset of patients recruited in Basel to ensure a stringent diagnosis of vascular dysregulation (n=264).

### DNA isolation

Genomic DNA was isolated from whole blood using the QIAamp DNA Blood Mini Kit (Qiagen, Hilden, Germany) according to the manufacturer’s instructions. Genotyping of each individual was conducted at least twice to verify results.

### Genotyping for the G894T polymorphism of *NOS3* and for the C242T polymorphism of *CYBA*

The G894T polymorphism of the human *NOS3* gene and the C242T polymorphism of the human *CYBA* gene were determined using the LightCycler^®^ FastStart DNA Master Hybridization Probes Kit (Roche Molecular Biochemicals, Mannheim, Germany), as described previously [35]. This method allowed rapid and reliable detection of single nucleotide polymorphisms by combining rapid-cycle polymerase chain reaction (PCR) and fluorescent melting point analysis. Single nucleotide polymorphisms were detected by differences in the melting temperature of fluorescent sequence-specific hybridization probes bound to the PCR product. Unlabeled primers for amplification were synthesized by MWG-Biotech AG (Ebersberg, Germany). Hybridization probes were designed and synthesized by TIB MOLBIOL (Berlin, Germany).

### Genotyping for the T-786C polymorphism of *NOS3*

The *NOS3* T-786C polymorphism was determined by PCR-restriction fragment length polymorphism (PCR-RFLP) as described previously [36]. Briefly, the 163 bp PCR product was purified with the QIAquick^®^PCR Purification Kit (Qiagen, Hilden, Germany) and digested with the restriction enzyme PdiI (MBI Fermentas, St. Leon-Rot, Germany). PdiI recognizes the mutant allele (C at position −786) and cleaves the 163 bp product in two fragments of 81 and 82 bp. The digestion products were size-separated by agarose gel electrophoresis (2%, stained with GelStar^TM^; Cambrex Bio Science Inc., Rockland, ME). The PCR product of an individual carrying two wild type alleles (TT) digested with PdiI revealed no fragments, whereas digestion of an individual homozygous for the C allele resulted in two fragments, which appeared as one band on the agarose gel, due to their similar size. Digestion of the PCR product of a heterozygous individual (TC) resulted in three fragments (163, 81, and 82 bp; visible as two bands).

### Statistical analysis

Age differences between the groups were evaluated using the unpaired Student *t*-test. Gender frequency was evaluated by Fisher’s exact test. Genotype, allele, and haplotype frequencies were compared by the χ^2^ test. Differences were considered significant when p<0.05. All probability values were based on two-tailed tests. Power analysis was conducted using Graph Pad StatMate version 1.01 (Graph Pad, San Diego, CA). This study had a power of 80% to detect a difference of 0.14 in the allele frequency for G894T (risk variant TT or GT) and T-786C (risk variant CC or CT), and a difference of 0.09 in the allele frequency for C242T (risk variant CC or CT).

## Results

The genotypes of 300 HTG patients and 127 NTG patients were assessed. Demographic data of the patients are summarized in Table 1. All participants were of Caucasian descent. There were significantly more women than men in the NTG cohort (p=0.0038). From the cohort recruited in Basel, 64 patients had a vascular dysregulation and 200 patients had no evidence of vascular dysregulation.

**Table 1:** Characteristics of the participants.

**Parameter**	**NTG (n=127)**	**HTG (n=300)**	**Fisher’s exact test p-value**
Age [years; mean±SD]	61±14	64±14	0.031
Male [n (%)]	41 (32.3)	143 (47.7)	
Female [n (%)]	86 (67.7)	157 (52.3)	0.0038
Patients with vascular dysregulation (from subset of n=264 patients) [n (%)]	22 (19.8%)	42 (36.6%)	0.004

### C242T polymorphism of *CYBA*

One NTG participant could not be unequivocally genotyped for the C242T polymorphism of the *CYBA* gene and was excluded from the statistical analysis. Table 2 shows the genotype and allelic distribution among normal and high tension glaucoma patients. There were no significant differences between the two glaucoma groups with respect to the distribution of alleles and genotypes and patients with and without vascular dysregulation were similar (Table 3). There were no significant differences by gender.

**Table 2:** Genotypic and allelic frequencies of the *CYBA* C242T polymorphism in normal (NTG) and high tension glaucoma (HTG) patients.

**Genotypes**	**NTG (n=126)**	**HTG (n=300)**	**χ^2^ test p-value**
CC [n (frequency)]	55 (0.44)	140 (0.47)	
CT [n (frequency)]	58 (0.46)	118 (0.39)	0.35
TT [n frequency)]	13 (0.10)	42 (0.14)	
**Alleles**			
C [n (frequency)]	168 (0.67)	398 (0.66)	0.93
T [n (frequency)]	84 (0.33)	202 (0.34)	

n=total number of patients for genotype, or total number of alleles (i.e., 2 alleles per patient).

**Table 3:** Genotypic and allelic frequencies of the *CYBA* C242T polymorphism in patients with and without vascular dysregulation.

**Genotypes**	**With vascular dysregulation (n=64)**	**Without vascular dysregulation (n=197)**	**χ^2^ test-value**
CC [n (frequency)]	32 (0.50)	91 (0.46)	
CT [n (frequency)]	24 (0.38)	85 (0.43)	0.72
TT [n frequency)]	8 (0.12)	21 (0.11)	
**Alleles**			
C [n (frequency)]	88 (0.69)	267 (0.68)	0.84
T [n (frequency)]	40 (0.31)	127 (0.32)	

n=total number of patients for genotype, or total number of alleles (i.e., 2 alleles per patient).

### G894T polymorphism of *NOS3*

The genotype distributions of the G894T polymorphism of the *NOS3* gene among patients with normal and high tension glaucoma and among patients with and without vascular dysregulation (i.e., vasospasm) are shown in Table 4 and Table 5. There were no statistically significant differences for the distribution of the two alleles and three genotypes by glaucoma type, vascular dysregulation, or gender.

**Table 4:** Genotypic and allelic frequencies of *NOS3* G894T polymorphism in NTG and HTG patients.

**Genotypes**	**NTG (n=127)**	**HTG (n=300)**	**χ^2^ test p-value**
GG [n (frequency)]	56 (0.44)	139 (0.46)	
GT [n (frequency)]	57 (0.45)	125 (0.42)	0.82
TT [n (frequency)]	14 (0.11)	36 (0.12)	
**Alleles**			
G [n (frequency)]	169 (0.67)	403 (0.67)	0.86
T [n (frequency)]	85 (0.33)	197 (0.33)	

n=total number of patients for genotype, or total number of alleles (i.e., 2 alleles per patient) .

**Table 5:** Genotypic and allelic frequencies of *NOS3* G894T polymorphism in patients with and without vascular dysregulation.

**Genotypes**	**With vascular dysregulation (n=64)**	**Without vascular dysregulation (n=200)**	**χ^2^ test p-value**
GG [n (frequency)]	22 (0.34)	88 (0.44)	
GT [n (frequency)]	34 (0.53)	87 (0.44)	0.36
TT [n (frequency)]	8 (0.13)	25 (0.12)	
**Alleles**			
G [n (frequency)]	78 (0.61)	263 (0.66)	0.32
T [n (frequency)]	50 (0.39)	137 (0.34)	

n=total number of patients for genotype, or total number of alleles (i.e., 2 alleles per patient) .

### T-786C polymorphism of *NOS3*

The T-786C polymorphism of the *NOS3* gene could not be determined unambiguously in two individuals (1 NTG, 1 HTG), and they were excluded from this analysis. Genotype and allelic distributions for NTG and HTG patients, as well as for patients with and without vascular dysregulation are shown in Table 6 and Table 7, respectively. There were no significant differences between the groups with respect to the distribution of the two alleles and three genotypes, by glaucoma type, vascular dysregulation, or gender. All of the above genotype and allele distributions fit the Hardy–Weinberg equilibrium.

**Table 6:** Genotypic and allelic frequencies of *NOS3* T-786C polymorphism in NTG HTG patients.

**Genotypes**	**NTG (n=126)**	**HTG (n=299)**	**χ^2^ test p-value**
TT [n (frequency)]	45 (0.36)	102 (0.34)	
TC [n (frequency)]	62 (0.49)	143 (0.48)	0.76
CC [n (frequency)]	19 (0.15)	54 (0.18)	
**Alleles**			
T [n (frequency)]	152 (0.60)	347 (0.58)	0.54
C [n (frequency)]	100 (0.40)	251 (0.42)	

n=total number of patients for genotype, or total number of alleles (i.e., 2 alleles per patient).

**Table 7:** Genotypic and allelic frequencies of *NOS3* T-786C polymorphism in patients with and without vascular dysregulation.

**Genotypes**	**With vascular dysregulation (n=64)**	**Without vascular dysregulation (n=197)**	**χ^2^ test p-value**
TT [n (frequency)]	20 (0.31)	64 (0.32)	
TC [n (frequency)]	30 (0.47)	96 (0.49)	0.86
CC [n (frequency)]	14 (0.22)	37 (0.19)	
**Alleles**			
T [n (frequency)]	70 (0.55)	224 (0.57)	0.67
C [n (frequency)]	58 (0.45)	170 (0.43)	

n=total number of patients for genotype, or total number of alleles (i.e., 2 alleles per patient).

### Haplotypes

Analysis of the prevalence of high risk haplotypes, i.e., homozygosity for at least two risk alleles (TT for G894T, CC for T-786C, and CC for C242T), was similar in patients with NTG and HTG (p=0.26).

## Discussion

Ocular perfusion plays a major role in glaucoma [2] and evidence for involvement of the NO system is considerable [37,38]. As an example, a study comparing the perfusion of the optic nerve head and fundus pulsation amplitude during NOS3 inhibition found a significantly lower perfusion and pulsation amplitude in HTG patients compared to controls, suggesting an altered NOS3 activity in these patients [39]. We therefore postulated that variants in genes involved in NO regulation could alter susceptibility to glaucoma in general and particularly in subgroups with evident vascular dysfunction. For our analysis we selected *NOS3* and *CYBA* polymorphisms that have previously been associated with vascular disease and therefore determined the distribution of G894T and T-786C in *NOS3* and C242T in *CYBA* in a large Caucasian glaucoma cohort. However, these polymorphisms had no different distribution between HTG and NTG patients and the presence of vasospasms had no impact.

The mutant allele of the G894T polymorphism of *NOS3*, which results in a glutamate to aspartate conversion at codon 298 (Glu298Asp) [40], has a higher prevalence in several cardiovascular diseases [24,40-46] as well as other diseases with endothelial dysfunction such as diabetic nephropathy [47] and frontotemporal lobar degeneration [48]. However, our study distribution of the polymorphism in glaucoma patients was similar and also comparable to the frequencies in healthy Caucasian populations [47,49-52].

The T-786C polymorphism in the promoter region of *NOS3* has also been repeatedly associated with cardiovascular disease [46,53-55]. Our analysis demonstrated a similar distribution of the polymorphism in both Caucasian glaucoma groups that was also similar to frequencies reported in healthy Caucasians [51,52,56].

The relationship between *NOS3* polymorphisms and glaucoma in Caucasians is poorly understood. An earlier study found that a *NOS3* variant in the promoter region close to the functional T-786C variant was positively associated with POAG [32]. Another study demonstrated no significant difference in the distribution of the *NOS3* repeat alleles in patients with glaucoma and controls and between HTG and NTG patients [33]. Interestingly, there was a significant difference in the distribution of *NOS3* allelic haplotypes (including T-786C) in patients with glaucoma and a history of migraine compared to controls suggesting some association with vascular dysfunction in glaucoma patients [33]. To date, the polymorphisms addressed in this study all associated with vascular disease, have either not been thoroughly investigated in Caucasian glaucoma populations, or often in smaller groups, and only rarely in NTG [33]. In agreement with our findings, a recent study in Caucasians found no difference in the distribution of several *NOS3* polymorphism (including G894T and T-786C) when POAG patients were compared with controls [34]. What our study further suggests is that also NTG patients and particularly those with clinically evident vascular dysfunction do not more likely carry these polymorphisms.

Studies in other ethnic groups mainly investigated other polymorphisms of *NOS3* and are therefore not readily comparable with our findings [57-59]. In a Chinese population, the single nucleotide polymorphisms rs3793342, rs743507, rs11771443, rs7830, and rs3918188 were not associated with POAG or with primary angle-closure glaucoma (PCAG). However, following haplotype-based case-control analysis, the frequency of the C-T haplotype established by rs3793342 and rs11771443 was significantly higher for POAG patients compared to controls, suggesting that *NOS3* may contribute to the occurrence of POAG in the Han Chinese population [57]. In a Pakistani cohort, a polymorphism in intron 4 of *NOS3* was associated with POAG and PCAG [58] while a Taiwanese case-control study reported no association of the distribution of this polymorphism and T-786C with POAG [59].

Another way to modulate local availability of NO is to modify its degradation and clearance. Hence, in contrast to *NOS3* polymorphisms, which may modify NO formation, the C242T polymorphism in *CYBA* reduces vascular NAD(P)H oxidase activity and thus local superoxide anion formation [14] thereby decreasing NO inactivation through this pathway. The 242T-allele could therefore represent a protective factor against vascular dysregulation in glaucoma. However, in the present study, the distribution of this polymorphism was also similar to the distribution described in other Caucasian populations [17,22,36,51] and in glaucoma types. Moreover, the combination of polymorphisms favoring NO deprivation was not different between the glaucoma populations.

This study has limitations. First, the study did not directly compare the *NOS3* polymorphism distribution of glaucoma patients with sex- and age-matched controls, but rather relied on previously published distributions in the Caucasian population for comparison. However, given the large number of participants with glaucoma in our study, it is likely that a clinically significant difference in rates of polymorphism in glaucoma would have been observed, if it even existed. Second, the subset of NTG with well characterized vascular dysregulation was smaller and statistical power was somewhat less. Third, the great majority of patients referred to the tertiary centers for treatment were recruited, had advanced disease. As this study was planned in a cross-sectional manner, no follow up visits were planned and made. Therefore the study does not answer the question of whether there is a relationship between the polymorphisms investigated and progression of glaucoma. Fourthly, since systemic and ocular treatments as well as their combination were numerous and varied from patient to patient, an analysis of the drugs has not been performed also in consideration that this was not part of the study outcome measures.

In conclusion, in a large cohort of Caucasian glaucoma patients we have studied the frequencies of a set of polymorphisms of the NO system, which all have previously been associated with cardiovascular disease, and have found no association between the frequencies of the polymorphisms and glaucoma. These results therefore suggest the absence of a relevant relationship between the tested polymorphisms and open-angle glaucoma in Caucasians.
